# The heterogeneity in link weights may decrease the robustness of real-world complex weighted networks

**DOI:** 10.1038/s41598-019-47119-2

**Published:** 2019-07-23

**Authors:** M. Bellingeri, D. Bevacqua, F. Scotognella, D. Cassi

**Affiliations:** 1 Dipartimento di Scienze Matematiche, Fisiche e Informatiche, Università di Parma, via G.P. Usberti, 7/a, 43124 Parma, Italy; 2 PSH, UR 1115, INRA, 84000 Avignon, France; 3 Dipartimento di Fisica, Politecnico di Milano, Piazza Leonardo da Vinci 32, 20133 Milano, Italy; 4 Center for Nano Science and Technology@PoliMi, Istituto Italiano di Tecnologia, Via Giovanni Pascoli, 70/3, 20133 Milan, Italy

**Keywords:** Computational biology and bioinformatics, Computer science, Complex networks

## Abstract

Here we report a comprehensive analysis of the robustness of seven high-quality real-world complex weighted networks to errors and attacks toward nodes and links. We use measures of the network damage conceived for a binary (e.g. largest connected cluster *LCC*, and binary efficiency *Eff*_*bin*_) or a weighted network structure (e.g. the efficiency *Eff*, and the total flow *TF*). We find that removing a very small fraction of nodes and links with respectively higher strength and weight triggers an abrupt collapse of the weighted functioning measures while measures that evaluate the binary-topological connectedness are almost unaffected. These findings unveil a problematic response-state where the attack toward a small fraction of nodes-links returns the real-world complex networks in a *connected but inefficient state*. Our findings unveil how the robustness may be overestimated when focusing on the connectedness of the components only. Last, to understand how the networks robustness is affected by link weights heterogeneity, we randomly assign link weights over the topological structure of the real-world networks and we find that highly heterogeneous networks show a faster efficiency decrease under nodes-links removal: i.e. the robustness of the real-world complex networks against nodes-links removal is negatively correlated with link weights heterogeneity.

## Introduction

The robustness of a network is its ability to maintain the system functioning in case of failures of nodes or links. Networks robustness is extremely important and has been widely investigated in last years in different fields of science^1–13^. A comprehensive analysis of network robustness considers the failure of both nodes (e.g. Iyer *et al*.^9^) and links (e.g. Pajevic and Plenz^13^). Initially, large attention has been dedicated to binary-topological analyses where the links among nodes are only present or absent. Yet, recent studies evidenced that the robustness of complex networks can be comprehensively understood only when considering the strength (weight) of the relationship (link) among nodes^13–22^.

The analysis of the robustness of complex weighted networks provided fundamental outcomes. Past studies demonstrated that when the network robustness is measured using the largest connected cluster (*LCC*), it is highly vulnerable to the removal of links with lower weight (weak links) but robust to deletion of links of higher weight (strong links)^18–21^. The widely accepted outcome was that ‘weak links are the universal key for complex networks stability’^21^. On the other hand, Pajevic and Plenz^13^ outline how the average clustering of nodes (that can be viewed as a measure of the local efficiency of the system) is robust to the removal of weak links but rapidly destroyed when removing links with higher strength. Dall’Asta *et al*.^22^ showed that introducing the weight of links in the US airports network would decrease its robustness with respect to classic topological frameworks. Further, Bellingeri and Cassi^23^ outlined how the network robustness response to node attacks changes according to the considered measures of the system functioning, i.e. weighted or binary.

In the present work, we investigate the role of the weighted structure of complex networks in shaping their robustness against the removal of both nodes and links. We analyze a high quality set of real-world weighted complex networks from different fields of science (Table 1). The considered network present different number of nodes, links, and a sound interpretation of the nature of link weights, e.g. in the US airports network, the weight identifies the passengers flowing from two airports; in the neural network of the nematode *C. Elegans*, it identifies the number of connections between two neurons. We randomly removed nodes or links to simulate an error in the system, and we eliminated nodes with higher number of links and with higher strength (e.g. higher sum of link weights) and links with higher weight to simulate an attack. This is the so-called *attack strategy* with nodes or links removed according to some structural properties of the network^1–9^. We evaluated the robustness of the complex networks to removal of nodes or links in terms of the decrease of network functioning measures reflecting both the binary-topological and the complex weighted structure of the system^3,7,8,14^. We therefore used the largest connected cluster in the network (*LCC*)^1–3^ and the unweighted efficiency (*Eff*_*bin*_)^14,15,23^, to get a binary (unweighted) measures of the network functioning and we used the efficiency (*Eff*)^14,15,23^ and the total flow in the system (*TF*)^17^ to have a weighted measures for the functioning of the networks (Table 2).

**Table 1:** Real-world complex networks features. N number of nodes; L number of links; <*k*> average node degree; <*w*> average node strength; <*E*_*w*_> average link weights.

Name	US Airports	*C. Elegans*	Human brain	Cargo ship	*E. Coli*	Netscience	UK Faculties
Type	Transport	Biological	Biological	Transport	Biological	Social	Social
Nodes	Airports	Neurons	Brain Regions	Ports	Metabolites	Scholars	Individuals
Weights	Passengers	Connections number	Connections density	Shipping journeys	Common reactions	Common papers	Friendship Strength
*N*	500	279	501	834	1100	1589	81
*L*	2980	2287	6038	4349	3637	2743	817
<*k*>	11.9	15.3	24.1	10.4	6.6	3.45	20.2
<*w*>	1815657	57.6	0.23	897.44	9.01	1.5	92.1
<*E*_*w*_>	152320.2	3.76	0.01	97.70	1.36	1.43	4.57
Ref.	^27^	^30, 31^	^32, 33^	^24^	^24, 28^	^34^	^35^
Key	Air	Eleg	Human	Cargo	Coli	Net	UK

**Table 2:** List of the complex network functioning measures used in this research.

Measure	Formula	Functioning meaning	Ref.
*LCC*	$LCC=\,\max ({S}_{j})$ *S*_*j*_: size of the *j* cluster	The *LCC* is the largest number of nodes connected by at least one path in the network and can be viewed as a binary (unweighted) measure of the network functioning.	^1– 3^
*Eff*	$Eff=\frac{1}{N\cdot (N-1)}\sum _{i\ne j\in G}\frac{1}{d(i,j)}$ *N*: number of nodes *D*_*i,j*_: length of the weighted shortest path between node *i* and *j*	The efficiency (*Eff*) is a measure of the global complex network capacity to deliver information among system constituents (nodes) and allows a precise quantitative evaluation of the weighted networks functioning. It is computed considering the link weights.	^13, 14, 22^
*Effbin*	$Effbin=\frac{1}{N\cdot (N-1)}\sum _{i\ne j\in G}\frac{1}{d(i,j)}$ *N*: number of nodes *D*_*i,j*_: length of the binary shortest path between node *i* and *j*	The efficiency (*Eff*_*bin*_) is a measure of the global complex network capacity to deliver information among system constituents (nodes). It is computed over the binary network, where all link weights equals 1.	^13, 14, 22^
*TF*	$TF=\sum _{i=1}^{N}\sum _{j=1}^{N}{w}_{i,j}$	The total flow in the system (*TF*) is the sum of the link weights; it represents the simplest weighted measure evaluating the actual or the potential flows between couple of nodes in the networks.	^17^

## Results and Discussion

**Figure 1:**
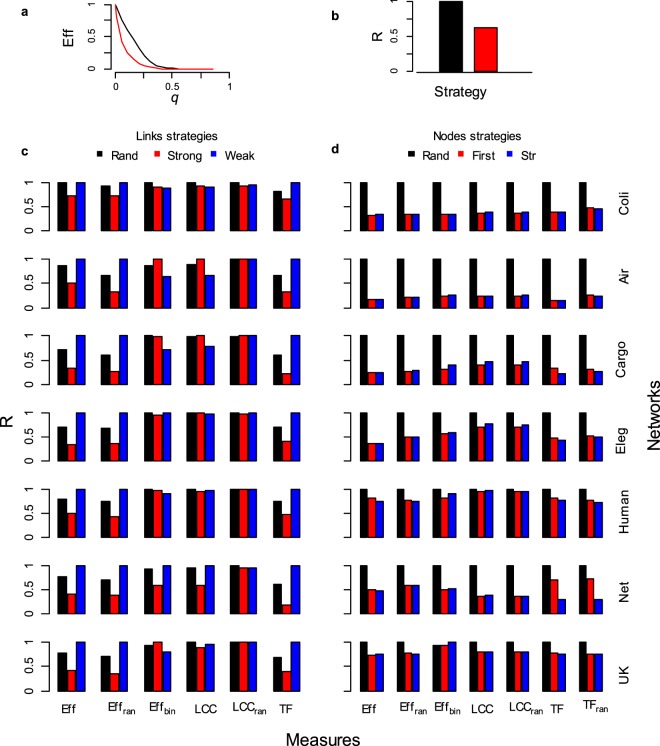
Robustness of seven real-world complex weighted networks under different strategies of nodes-links removals. The robustness of real-world complex weighted networks was analyzed under different nodes-links removals strategies and using different measures for the system functioning. (**a**) Efficiency of the system (*Eff*) as a function of the fraction of nodes or links removed (*q*). The network efficiency (*Eff*) decreases under nodes-links removal; in this example the red strategy produced a sharper decrease in the network efficiency meaning that is more harmful than the black strategy. (**b**) Example of the complex networks robustness (*R*) for the two strategies of nodes or links removal of the outcomes in (**a**). The robustness (*R*) of the removal strategy is the area below the curve produced by the removal strategy in (**a**). The robustness (*R*) produced by the removal strategy is normalized on the max value of the strategy robustness for that system functioning measurement; in this way we can easily compare the robustness of the network under different nodes-links removal strategies. (**c**,**d**) The real-world networks robustness (*R*) under different nodes-links removal strategies. (**c**) Links removal strategies. (**d**) Nodes removal strategies. *Eff* indicates the weighted system efficiency computed on the real-world networks; *Eff*_*ran*_ is the weighted efficiency computed on the real-world network after the randomization of the link weights, i.e. the real link weights are randomly re-assigned on the network links. *LCC* indicates the largest connected cluster in the network; *LCC*_*ran*_ is the largest connected cluster measurements computed on the real-world network after the randomization of the link weights; *TF* is the total flow in the network, i.e. the sum of the all link weights; *TF*_*ran*_ is the total flow on the real-world networks after the randomization of link weights. The random removal (*Rand*) and the randomized link weights network counterparts (*LCC*_*ran*_, *EFF*_*ran*_ and *TF*_*ran*_) outcomes are the average of 10^4^ simulations.

When links or nodes are removed from the network we can assess the decrease of the system functioning according to different measures as showed in Fig. 1a. The more important are the components removed from the network, the steeper is the decrease in the network functioning measure. For example, in Fig. 1a the red removal strategy identifies more important components in the network, since a given fraction *q* of nodes-links removed, triggers a steeper decrease in the network functioning efficiency (*Eff*, normalized on the initial maximal value) with respect to the black strategy. To compare the response among networks and measures, we resume the removal outcomes in a single value defined as the network robustness (*R*), reported in Fig. 1b. The value of *R* corresponds to the area below the curve of the system functioning against the fraction of nodes-links removed and ranging between two theoretical extremes, *R* ≃ 0 and *R* ≃ 1. In Fig. 1 we show the robustness outcomes under different types of nodes-links removals.

### Links removal

When quantifying the system functioning with *Eff* we find the real-world complex networks to be highly vulnerable to the removal of links with higher weight (Fig. 1c); i.e. *Strong* strategy produces the fastest decrease of the system efficiency functioning (*Eff*). Further, we found real-world complex networks *Eff* to be very robust to the deletion of weaker links and *Weak* links removal strategy is highly ineffective even causing an efficiency decrease lower than the random removal of links (*Rand*). At the opposite, when measuring the network functioning with *LCC*, we find *Strong* and *Weak* links attack strategies the less and the most effective to reduce the *LCC*, especially for Cargo and Air networks (Fig. 1c). Only in the social networks (Net and UK), the deletion of strong links is able to vanish the *LCC* faster than *Weak* links removal. This confirms recent analyses showing that the deletion of strong links preserves the *LCC* until the weakest links are removed^18–21^, except for the real-world social networks under study.

**Figure 2:**
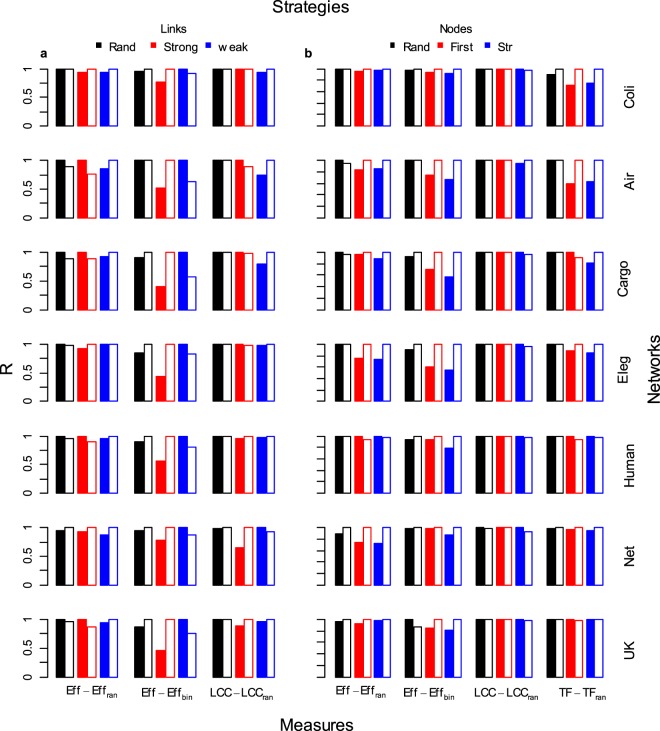
Robustness of seven real-world complex weighted networks with real and randomized link weights. Real-world complex weighted networks was analyzed comparing the robustness (*R*) of real and randomized link weights. In this figure we show the robustness (*R*) comparing outcomes of the real-world network with the randomized version of the same system. In the randomized network weights are reshuffled and randomly reassigned over the links; in this manner the binary-topological structure is maintained. The filled box indicates the robustness of the real-world system and the empty box of the same color contour shows the robustness of the randomized version of the same system. (**a**) Links removal strategies. (**b**) Nodes removal strategies. *Eff* indicates the weighted system efficiency computed on the real-world networks; *Eff*_*ran*_ is the weighted efficiency computed on the real-world network after the weights reshuffle, i.e. the real link weights are randomly re-assigned over the network. *LCC* indicates the largest connected cluster in in the network; *LCC*_*ran*_ is the largest connected cluster measures computed on the real-world network after the randomization of the weights; *TF* is the total flow in the network, i.e. the sum of the all link weights; *TF*_*ran*_ is the total flow computed on the real-world networks after the randomization of link weights. For the links removal strategy *TF* is not plotted because the real and the randomized link weights return the same hierarchy of links removal and thus the same outcomes of system total flow decrease. The random removal (*Rand*) and the randomized link weights network counterparts (*LCC*_*ran*_, *EFF*_*ran*_ and *TF*_*ran*_) are the average of 10^4^ simulations.

**Figure 3:**
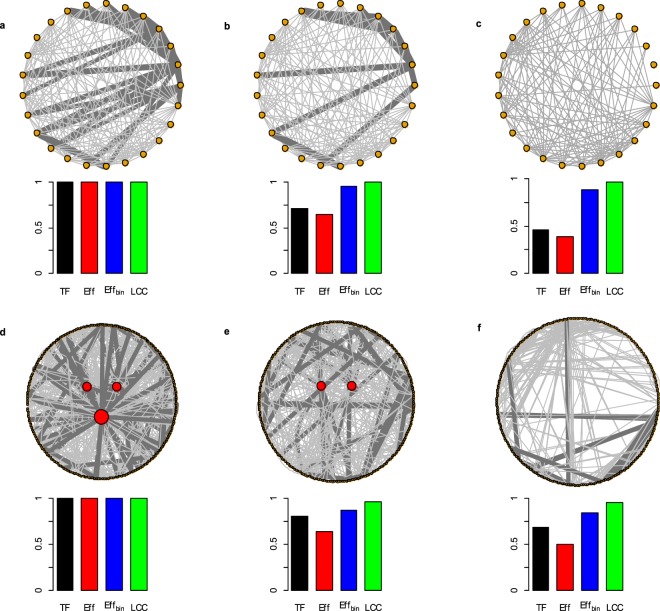
Abrupt functioning efficiency collapse and the ‘connected but inefficient’ network state. Step by step strong links removal simulation on a sub-network (N = 25) of the US Airports and three nodes removal on the *C. Elegans* real-world complex weighted network. The total flow *TF* and the weighted efficiency *Eff* readily decrease under the removal of links with higher weight and nodes with higher strength. (**a**) Initial sub-network of 25 nodes and 153 links drew from US Airports network. The weight of the links identifies the number of passengers flowing from the connected airports; the weights ranges from a minimum of 9 to maximum of 2.253.992 passengers per year. For the illustrative example we maintain only the strongest links with weight >10^6^ (26 strongest dark grey links) and <380.000 passengers per year (127 weakest soft grey links). (**b**) The sub**-**network after the removal of 16 links of highest weight (10% of the links). (**c**) The sub**-**network after the removal of 26 links of highest weight (17% of the links). (**d**) *C. Elegans* real-world complex weighted network representing nodes-neurons and the links connections number among them (link weights). Dark grey are the strongest and soft grey are the weakest links. The three red nodes of higher strength are outlined in the center where the others nodes are in the circle layout. (**e**) *C. Elegans* network after the removal of the first highest strength node. (**f**) Neuronal network of *C. Elegans* following the removal of the three main nodes.

In real-world networks, link weights are coupled to the binary topology in a non-trivial way^18–22^, for instance with nodes strength-degree correlation meaning that links with higher weight are more likely joining high degree nodes^16,17^. For this reason it is important to understand if strong links are important in supporting *Eff* because of their large weight or of their specific occurrence among more connected nodes (hubs) of the network. We compute the robustness of the real-world networks after weights randomization over the topological structure, with link weights independent of any topological features and acting as a control outcome. *Strong* strategy results to be the most harmful to decrease *Eff* even when randomizing the real weights over the binary-topological structure, i.e. the importance of strong links to support *Eff* is maintained also when the real weight-topology coupling is not correlated (Fig. 1c, see *Eff*_*ran*_). We then perform an *experimentum crucis* by removing links according to the real link weights, then measuring *Eff*_*bin*_ considering links like binary (all weights equals to 1). In this way we nullify the influence of the weight to shape the efficiency (*Eff*) and we maintain only the binary-topological role of the strong links. We find an *efficiency reversal pattern* for all the real-world complex networks where weak links removal (*Weak*) readily decreases the efficiency functioning whereas *Strong* becomes ineffective (Fig. 2a, *Eff* − *Eff*_*bin*_ column). In other words, the importance of strong links to support the information delivery efficiency is mainly due to their larger intensity, with a secondary role of their topological positioning. These results bring important evidences inside the long standing debate about the importance of weak and strong links^13,18–21,24^ showing that links carrying larger weight would be fundamental to support the efficiency of the system hence not being the main responsible of the topological connectedness of the network. We also revise the importance of weak links in support network robustness confirming their function in maintaining the topological connectedness of the network^18–21^ but also their small relevance to the information delivery efficiency (Figs 1c, 2a). Very important, we outline that removing a small fraction of strong links can readily reduce the real-world network information delivery efficiency (*Eff*) despite the size of the largest connected cluster (*LCC*) is still preserved. In other terms, the binary network functioning parameters *Eff*_*bin*_ and *LCC* are constant under the removal of links with higher weight (strong links) whereas *Eff* and *TF* experience a fast decrease and leave the system in a ‘*connected but inefficient’* network state. For example, removing a small fraction of busy shipping routes (10% of the strong links) from Cargo-ship network produced a quick collapse in the system efficiency (losing 50% *Eff*) while isolating only 2% of ports-nodes (2% *LCC*) (Fig. S8, *Strong* row). Further, in *C. Elegans* network the removals of 10% of strong links induced the 60% *Eff* decrease yet leaving roughly unaltered the size of the *LCC* (Fig. S8, *Strong* row). This discover outline an under-appreciated but most likely pattern of network failures: namely that most failures may occur even though the network is completely connected. For the sake of an example, we illustrate this finding in Fig. 3a–c for a subgraph of the US Airports network. After the removals of 16 links of highest weight, i.e. 10% of the links (Fig. 3b), the system loses 29% of the total flow (*TF*, black bar) and the 35% of the system functioning efficiency (*Eff*, red bar) but only the 4% of the binary efficiency (*Eff*_*bin*_, blue bar) and no decrease in the largest connected cluster (*LCC*, green bar). After the removals of 26 links of highest weight (17% of the links), the total flow (*TF*) is the 44% of the initial flowing meaning that the removal of only 17% of the links is able to roughly halves the number of passengers from this subset of airports (Fig. 3c). Further, the system functioning efficiency (*Eff*) collapses to 38% of its initial efficiency. At this final step where all the 26 strongest links are removed, the network loses only the 12% of the binary efficiency (*Eff*_*bin*_) and only one node is disconnected from the network with a decrease in the largest connected cluster (*LCC*) of the 4%.

### Nodes removal

**Figure 4:**
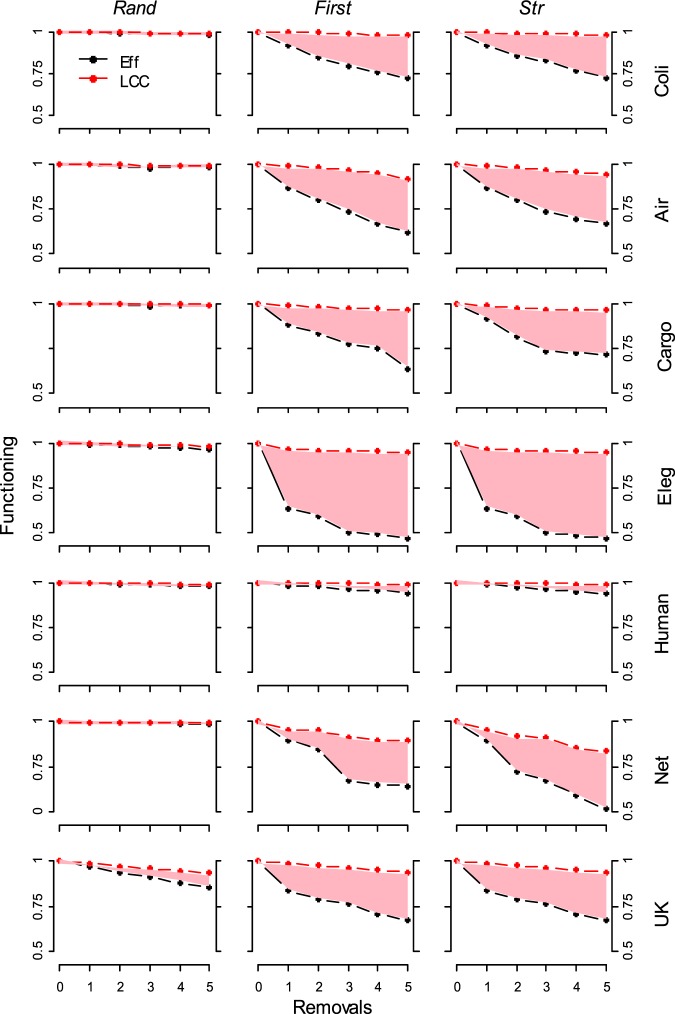
Real-world complex weighted networks functioning decrease (*Eff* vs *LCC*) under few removals. The system functioning is normalized by the initial functioning value (e.g. before any removal). The pink area depicts the difference between *Eff* and *LCC* measures along the nodes removal process. For all systems except Human brain network, after very few (1 to 5) higher strength-degree nodes removal we observe a quick efficiency (*Eff*) decrease whereas the largest connected cluster (*LCC*) remains roughly constant.

We find that real-world networks are robust to the random removal but vulnerable to the deletion of higher connected and higher strength nodes for all the functioning measures, i.e. real-world weighted networks would be “*random resistance*” and “*attack prone*” (Fig. 1d) confirming classical binary-topological outcomes^1–9^. Very interesting, removing a few nodes abruptly collapse the functioning efficiency (*Eff*) of the networks (Fig. 4). For example, in Fig. 3d–f we depict the outcomes of the removal of the three highest strength nodes in the *C. Elegans* neuronal network. Removing the first highest strength node triggers the 27% of the efficiency (*Eff*) and the 20% of the total flow *TF* decrease, but only 3% of the *LCC* and 2% of the *Eff*_*bin*_. (Fig. 3e). Removing the three main nodes the *C. Elegans* is deprived of the links with higher weight (dark grey links) with a sharp decrease of the efficiency *Eff* (50%) and of the total flow *TF* (32%). Nonetheless, a multitude of weak interactions (soft grey links) holds the network still connected showing a minimal *LCC* (4%) and *Eff*_*bin*_ (5%) decrease (Fig. 3f). We find the same system vulnerability in the other real-word networks, for example removing 5 nodes-metabolites among the 1100 total nodes-metabolites in *E. Coli* network decreases the 30% of the efficiency and only the 2% the *LCC* (Fig. 4); in the US Airports network, 5 nodes-airports removals over the N = 500 airports sharply decreases the total flow (*TF*) to the 60% of the initial value with 5% *LCC* decrease (Fig. S10); further, 5 nodes-ports attack reduces to the 70% the efficiency in the Cargo-ship network (Fig. 4). Only for the Human brain network we find a small difference in the measurements with *Eff* close to the *LCC* (Figs 4 and S10). In all the networks the binary efficiency (*Eff*_*bin*_) follows the *LCC* with a slow decrease. These outcomes can be resumed in a novel and problematic response pattern of the real-world complex networks, where the removal of nodes-links playing a major role in the energy-information delivery may leave the system in a *connected but inefficient state* (Fig. 3).

Dall’Asta *et al*.^22^ showed that when removing highly connected nodes the total ‘outreach’ (e.g. a measure computing the product the link weight and the Euclidean distance covered by the link connecting airports) of the US Airports network decreased much rapidly than its *LCC* measure. Our findings wide Dall’Asta *et al*.^22^ outcomes for different kinds of network and measures able to evaluate its weighted structure, unveiling that even removing a few nodes can leave real-world complex weighted networks in a *connected* (the *LCC* and *Eff*_*bin*_ are preserved) *but inefficient state* (*Eff* and *TF* quickly collapse). Comparing the normalized *LCC* and *Eff* trends under few nodes removals, we observe narrow difference in the network functioning measurement, with a sharper decrease of the network efficiency *Eff*. This difference is quantified by the pink area between the curves in Fig. 4 for all the real-world complex networks. For example, for the Net scientist co-autorship network, after 5 higher strength nodes removals we find *Eff* collapsing below the 50% of the initial value where instead the *LCC* is around the 85% (Fig. 4, *Str* column). In the Coli network, 5 high degree nodes removals only, are able to decrease *Eff* below the 75% leaving the *LCC* roughly unchanged (Fig. 4, *First* column). These evidences outline how using binary measurements like the widely used largest connected cluster (*LCC*) may overestimate the robustness of real-world networks.

Very interesting, we find that real-world networks exhibit higher efficiency (*Eff*) robustness to the removal of nodes after the link weights randomization (except for the Human brain) (Fig. 2b, *Eff − Eff*_*ran*_, and Fig. S7). We find an important higher efficiency (*Eff*) robustness to nodes attack of the randomized networks for a few of nodes attack as well (Fig. S11). In networks with strength-degree correlation, the removal of higher connected nodes (the so-called hubs) will delete strongest links with higher information loss; differently, in the control network, the weights randomization eliminates the correlation and the removal of hubs would intercept less strong links with a reduced decrease in the information delivery efficiency. This finding indicates that some level of nodes degree-strength coupling discovered in real-world complex networks^16,17,22,24^ would make these systems even more vulnerable to nodes attack.

### The role of weights heterogeneity

**Figure 5:**
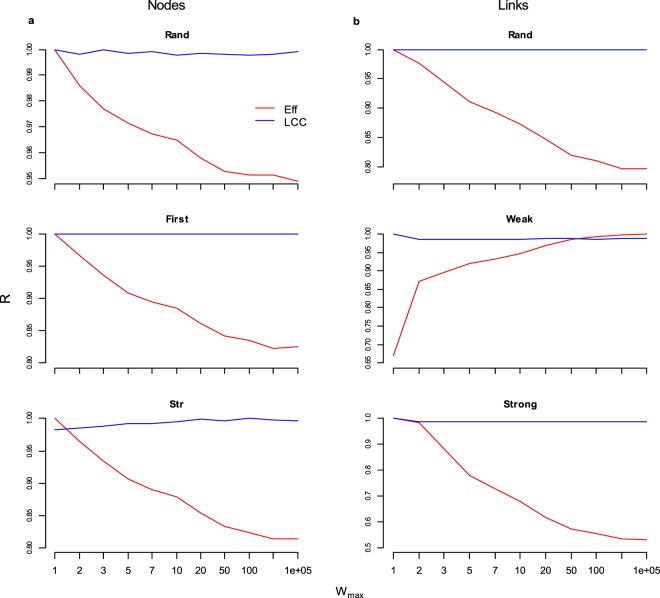
The real-world complex networks robustness of the efficiency functioning (*Eff*) decreases with links weight heterogeneity where instead the *LCC* is approximately constant. The robustness of the efficiency *Eff* and largest connected cluster *LCC* measures of the system functioning under nodes and links removal strategies. We randomly assigned the weight of the links over the real-world topological structure of the networks under exam. Link weights are sorted from 2 values distribution (1, *Wmax*); the upper limit *Wmax* ranges in (1, 10^5^); the outcomes are the average of 10^4^ simulations. For sake of example we depict the outcomes from the Cargo ship and the *E. coli* network. Left column**:** Cargo ship network under nodes removal strategies; Right column: *E. Coli* network under links removal strategies.

To examine in depth how link weights pattern influences the robustness of real-world networks we removed nodes-links increasing the link weights heterogeneity, by assigning link weights sorted from a rectangular and from a two values distribution (Supplemental Material S2). The link weights heterogeneity is enhanced by progressively increasing the maximum weight (*Wmax*). We find that the robustness of the network efficiency (*Eff*) is negatively correlated with link weights heterogeneity; differently, the robustness of the largest connected cluster (*LCC*) remains roughly constant by increasing the link weight heterogeneity (Fig. 5). This discovery would indicate that the overestimation of the network robustness adopting the *LCC* measure may be higher in real-world complex networks owing larger heterogeneity in the weights of the links.

**Figure 6:**
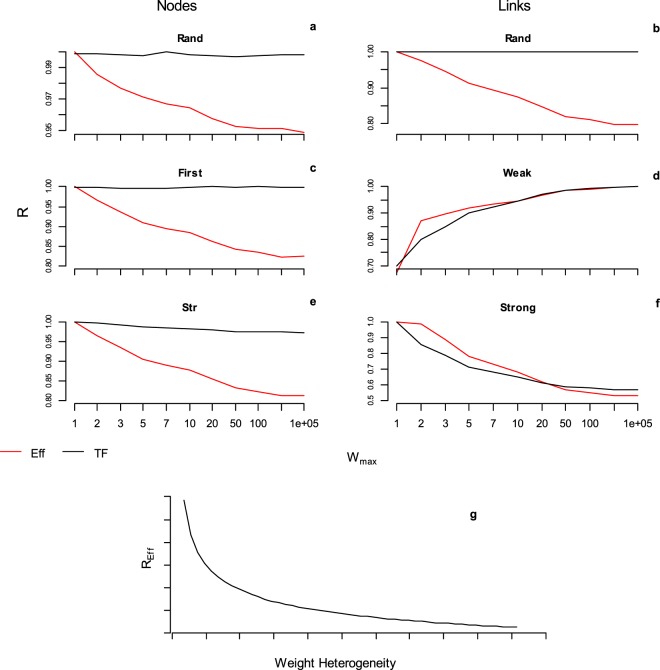
The real-world complex networks robustness of the efficiency functioning (*Eff*) decrease with link weights heterogeneity. The robustness of the efficiency *Eff* and total flow *TF* of the system functioning under nodes and links removal strategies. We randomly assigned the weight of the links over the real-world topological structure of the networks under exam. Link weights are sorted from 2 values distribution (1, *Wmax*); the upper limit *Wmax* ranges in (1, 10^5^) and for each *Wmax* value we performed 10^4^ simulations. For sake of example we depict the outcomes from the Cargo ship and the *E. coli* network. Left column**:** Cargo ship network under nodes removal strategies; Right column: *E. Coli* network under links removal strategies. In the bottom figure we exemplify the discovery outcomes: the robustness of the efficiency (*R*_*Eff*_) is negatively correlated with the heterogeneity of link weights, i.e. increasing the variance in the weight of the links, the real-world complex network become more vulnerable to nodes-links removal, both selective and random.

Surprisingly, increasing *Wmax* we assist to a decrease in the robustness (*Eff*) for all the nodes removal strategies (Fig. 6a) whereas the proportion of total flow (*TF*) subtracted to the networks remains roughly constant (Fig. 6a). For random links removal we find the same pattern (Fig. 6b). All these trends are more pronounced for the two values distribution of link weights (Fig. S11). This indicating that the decrease in robustness efficiency is not due to a major amount of ‘weight’ intercepted in the networks with increasing *Wmax*, but it would be an effect of the larger link weights heterogeneity (See Supplemental Material S3 for additional results and discussion). Differently, the total flow subtracted in the network by *Strong* and *Weak* links removal is clearly related to the weights heterogeneity, and we observe a robustness decrease for strongest (and an increase for weakest) links removal for both *TF* and *Eff* measures (Fig. 6b).

This last discovery of the negative relationship between network efficiency decrease and link weights heterogeneity shows that the robustness decrease is not only related to the transition from binary (*LCC*) to weighted measurement as suggested from Dall’Asta *et al*.^22^, but it is within a more general mechanism by which enhancing link weights heterogeneity negatively affects the robustness (*Eff*) in real-world complex networks (Figs 6, S12). The heterogeneity in link weights is interpreted as a feature able to stabilize different real-world networks^21,25^ with these systems self-organizing toward large heterogeneity in link weights (with many weak links). Our discoveries suggest that if the real-world complex networks are systems evolving toward larger link weights heterogeneity, they may pay the price in terms of robustness, with potential higher vulnerability to nodes-links failure.

## Conclusion

The problem of measuring the robustness of a network to targeted attacks or to random failures of its nodes-links components is rapidly becoming major topics of research in the complex network community^26^. This for the urgency to reduce the adverse consequences associated with real-world systems failures, and especially of critical infrastructures. The robustness of complex networks is mainly investigate focusing on the binary version of these systems. Our study showed that adopting measures of functioning able to evaluate the link weights structure strongly change the response of the real-world weighted networks under attack or failure. We find that the attack of a very small fraction of nodes-links can trigger an abrupt collapse of the weighted functioning measures while parameter that evaluate the simple binary connectedness are almost unaffected, i.e. the attack toward few nodes-links returns these systems in what we called a *connected but inefficient state*. This finding has important implications since the most of the real-world system failures may occur even though the network is completely connected and for this reason, adopting a binary measures of the network functioning like widely used the largest connected cluster (*LCC*) may overestimate the network robustness.

On the other hand, to investigate the reasons bearing the robustness of real-world networks is the gate to understand the mechanisms underlying evolution of these complex systems. Most of the studies focusing the binary structure of the real-world networks showed yet classical outcomes about the evolution of real-world networks, such as their small-world characteristic^1,7^, e.g. nodes are close each other in real-world networks, or their robustness to failure in spite of higher levels of vulnerability to the attack of higher connected nodes^1–3^. Analyses of weighted networks showed that links of lower weight play a key role in sustaining the connectivity of the system outlining the importance of weak links for real-world complex networks stability^21^. In this contribution we furnished a complementary perspective outlining the importance of links with higher weight for the real-world network functioning, by showing that a small fraction of strong links removal rapidly decreases the efficiency and the total flow in these systems.

Last, we investigated how the difference in the weight of the links affects the robustness of real-world networks. We find that the artificial increase of the link weights heterogeneity produced a faster efficiency (*Eff*) decrease under attack and failures. This finding open a new perspective showing that real-world networks with higher level of weight heterogeneity associated to the links may be less robust to attacks or failures.

Our study leads to several additional observations and interesting directions for future investigations. First, to analyze the robustness of real-world networks under attacks where nodes-links are ranked according to weighted properties, such as the weighted betweenness centrality, the weighted closeness centrality or others. To understand which attack based on weighted properties of the nodes is able to trigger the higher damage in the network will be a novel approach in the research investigating the nodes attack strategies efficacy. Second, to encourage studies exploring what we consider an important but under-appreciated fact of network failures: namely that most failures occur even though the real-world networks are completely connected. This can be the case of many daily life examples in transportation networks, such as a street closure in a road network or the personal strike closing nodes-airports in the flying network. But it can be also a frequent occurrence in biological and social networks, like the inhibition of some nodes-neurons functioning in brain networks or the leaving of individuals in working community. In all these examples from different reality domains, the network functioning is expected to decrease even though its general connectivity is still preserved. Last, we showed an interesting negative correlation with link weights heterogeneity and the robustness of real-world networks. Since real-world weighted networks owns significant level of heterogeneity in links weights^21,24,27,28^ our last result bring important implications for the robustness of the real systems. This result comes from numerical simulations based on seven given real networks. We hope it could inspire future theoretical investigations that might bring to more general mathematical proves of the observed relationship.

## Methods

### Nodes removal strategies

We follow 3 nodes removal strategies:

- *Rand*: nodes are randomly removed. This type of removal represents the possibility of failure (error) in the network^8,9^. For the random process of nodes removal (*Rand*) we averaged the outcomes from 10^4^ simulations.
- *First*: nodes are removed according to the degree of the nodes, i.e. the number of links to the node^8,9^. The number of the links to the node corresponds to the neighbors nodes number. *First* is the binary nodes removal strategy, not considering the weight of the links and it represents the classical network attack^8^.
- *Str*: nodes are removed according to the strength, i.e. the sum of the weight of the links to the node^23^. This is the weighted counterpart of the binary attack strategy *First*.

### Links removal strategies

We follow 3 links removal strategies:

- *Rand*: links are randomly removed. This type of removal represents the possibility of links failure (error) in the network^13^. For the random process of links removal (*Rand*) we averaged the outcomes from 10^4^ simulations.
- *Strong*: links are removed in decreasing order of weight, i.e. links with higher weight are removed first^13^ and it represents an attack directed to the links.
- *Weak*: links are removed in increasing order of weight, i.e. links with lower weight are removed first^13^.

The sequential removal of nodes-links is evaluated on the initial configuration of the network (i.e. before any removals) and not recalculated. In presence of ties (i.e. nodes or links with equals ranking properties) nodes-links with equals rank are randomly sorted.

### The measures of network functioning

#### The largest connected cluster (*LCC*)

The largest connected cluster (*LCC*) is a widely used measure of the network functioning^1–3^. The *LCC* is also known as giant component and it is the highest number of connected nodes in the network. Complex network may present different clusters, e.g. different subsets of nodes connected among them but disjointed with nodes belonging to other clusters. Let be *S*_*j*_ the size of the *j*-th cluster in the network, the *LCC* is the maximum cluster size:

1 $$LCC=\,\max ({S}_{j})$$

The removal of nodes-links may disconnect nodes belonging to the giant component thus producing the *LCC* measure decrease. The *LCC* decrease indicates that if we start from one node chose at random in the largest connected cluster, less nodes in the network are reachable. The *LCC* is a simple indicator evaluating the topological connectedness of the network and it not account the weight of the links that join nodes belonging to the largest connected cluster. We can see the *LCC* as an indicator of the possibility to reach nodes in the network with no consideration about the magnitude of the links-pathways we have to travel. For this reasons we adopt it like a binary measure of the network functioning not reflecting the heterogeneity of the link weights.

#### The total flow (*TF*)

The total flow is the sum of link weights and it represents the actual or the potential flowing between nodes pairs in the network. For example, in the transportation network of the US Airports the *TF* measure represent the actual flows between nodes pairs (where ‘actual’ means the flying passengers in a year); also in the transportation Cargo ship network *TF* represents the actual flow indicating the shipping journeys between ports in a year. Differently, in the Human brain network, where the weight of the link indicates the fiber connection density among brain regions, *TF* that is the sum of all the weights is a potential energy-information flowing along the fibers connecting nodes pairs, or in the *C. Elegans* real-world complex weighted network, *TF* indicates the total number of connections realized between pairs of neurons. In other terms, *TF* can be viewed as the thermodynamics capacity or a quantity influencing the actual flow between nodes pairs in the network but do not uniquely determine it, e.g. the higher is the connection density in the brain network, the higher can be the information delivered between brain regions in the unit of time.

Let be the weighted network G_w_ can be represented by a *N* × *N* matrix W where elements *w*_*ij*_ > 0 if there is a links of weight *w* between nodes *i* and *j, w*_*ij*_ = 0 otherwise.

The total flow is computed as:

2 $$TF=\sum _{i=1}^{N}\sum _{j=1}^{N}{w}_{i,j}$$

The total flow in the system (*TF*) is the sum of the link weights not reflecting the global weighted structure of network. In particular, *TF* not consider the links paths necessary to travel among nodes or the related global information delivery efficiency (like the *Eff* measures) or the nodes connectedness (as the *LCC*). Thus, when the network is subjected to the removal of nodes or links in our simulations, *TF* will not evaluate the following elongation of the paths among nodes or the nodes disconnections (or other combined changes) in the global network structure, but only it accounts the decrease in the total weight of the links. Since *TF* does not evaluate the global network structure we can see it like the simplest measure evaluating the weighted networks functioning.

#### The weighted efficiency (*Eff*) and the binary efficiency (*Eff*_*bin*_)

The concept of efficiency of the network was proposed by Latora and Marchiori^14^ with the aim to introduce a measurement encompassing the difference in link weights in the evaluation of the weighted networks functioning. The efficiency of a network is a measure of how efficiently it exchanges information. On a global scale, i.e. considering all the nodes-components of the system, the efficiency quantifies the exchange of information across the whole network where information is concurrently exchanged. The efficiency is a robust and widely used measure of the network functioning^14,15,23,29^.

The efficiency measurement is based on the shortest paths notion^17^. In network science, the shortest path is the path between two nodes in a network such that the sum of the weights of its constituent links is minimized. In a binary network a shortest path between a couple of nodes is an integer number computing the minimum number of links to travel from one node to the other. To compute the shortest path in a weighted network we have to account the difference in link weights and to do this we first compute the inverse of the link weights. This is a standard procedure^14,15,23,29^ with the aim to decrease the length of the links with higher weight and increase the length of the links with lower weight. In this way nodes joined by links with higher weight are more close to each other. This procedure has a straight meaning in real-world weighted networks. For example, in the *C. Elegans* network, two nodes-neurons joined by higher number of links-connections (higher weight) are closer than couples of neurons with lower number of connections (lower weight). For this reasons we can see the inverse of the link weights as a ‘weighted distance’ covered by the link and higher weight links as ‘shorter and faster routes’ for the information travelling. Thus, in a weighted network the shortest path between a couple of nodes is the minimum sum of the ‘weighted distances’ necessary to travel from the nodes. In the Supplemental Materials S1 we furnish a step by step example of the shortest paths computation in binary and weighted networks.

The efficiency of the network is then defined:

3 $$Eff=\frac{1}{N\cdot (N-1)}\sum _{i\ne j\in G}\frac{1}{d(i,j)}$$

where *N* denotes the total nodes in a network and *d(i, j)* denotes the length of the shortest path between a node *i* and another node *j*. When we include weight to the links (computing the weighted shortest paths) we obtain the weighted efficiency *Eff*; in the case we do not associate weight to the links (computing the simple binary shortest paths) we obtain the binary efficiency *Eff*_*bin*_. For a detailed explanation of the network efficiency measurement see Supplemental Materials S1. The efficiency here defined works with binary or weighted networks as well but it furnishes different interpretation of the information delivery in the system. *Eff*_*bin*_ considers the information delivery as all the network links owns equal capacity; differently, *Eff* precisely evaluates the effect of link weights heterogeneity to shape the shortest paths in the network and thus affecting the information delivery efficiency. We point out that the efficiency (*Eff*) and the total flow (*TF*) are measures able to account the heterogeneity in link weights with different meaning. The efficiency (*Eff*) is measure based on the shortest paths evaluating the global network structure capacity to deliver information among nodes and a decrease in the efficiency indicates a reduction in the energy-information pace exchanging over the network. The total flow (*TF*) is the sum of the link weights not accounting the shortest paths and the global network structure; thus a decrease in the total flow (*TF*) indicates a simple reduction in the link weights sum over the network. For example, supposing we remove some strong links in the networks, the *Eff* measure will evaluate both the higher weight lost in the system and the elongation of the shortest paths, whereas the *TF* will only evaluate the simple reduction in the link weights sum.

#### Real-world complex networks with modified link weights

We modify the weight of the links maintaining the binary topological structure of these systems. We first randomly assign to each link a weight sorted from the rectangular distribution tuning the sole parameter *Wmax* indicating the allowed maximum links weight. Then we randomly assign the weight to the links sorting from the bimodal distribution where links weight can have only two distinct values with equals probability (1, *Wmax*). For both the distributions, we tune *Wmax* with values {1, 2, 3, 5, 7, 10, 20, 50, 100, 1000, 100000}; for *Wmax* = 1 all the links have weight = 1 (higher network homogeneity) and the network is binary; for *Wmax* ≥ 2 the network starts to exhibit variance in link weights with the max variance for *Wmax* = 100000 (higher network heterogeneity). In this way the binary topological structure of the real-world network is maintained but any pattern of correlation between the topological and the weighted structure is eliminated, making possible to understand how the simple heterogeneity in links weights affects the system robustness. Since the link weights assignment is a random procedure, we perform 10^4^ simulations for each *Wmax* value.

## Supplementary information

Supplemental material
