# Supplementary material for: The heterogeneity in link weights may decrease the robustness of real-world complex weighted networks
Source: Sci Rep. 2019 Jul 23;9:10692. doi: 10.1038/s41598-019-47119-2 (PMC6650436; doi:10.1038/s41598-019-47119-2)
Supplement: Supplementary file 1 — Supplemental material [file 41598_2019_47119_MOESM1_ESM.docx]

**The heterogeneity in link weights may decrease the robustness of real-world complex weighted networks**

Bellingeri M.^1*^, Bevacqua D.^2^, Scotognella F.^3,4^, Cassi D.^1^

^1^Dipartimento di Fisica, Università di Parma, via G.P. Usberti, 7/a, 43124 Parma, Italy

* Corresponding author: [michele.bellingeri@unipr.it](mailto:michele.bellingeri@unipr.it)

^2^PSH, UR 1115, INRA, 84000, Avignon, France

^3^Dipartimento di Fisica, Istituto di Fotonica e Nanotecnologie CNR, Politecnico di Milano, Piazza Leonardo da Vinci 32, 20133 Milano, Italy

^4^Center for Nano Science and Technology@PoliMi, Istituto Italiano di Tecnologia, Via Giovanni Pascoli, 70/3, 20133, Milan, Italy

Supplementary materials

**S.1. The measures of network functioning**

**The weighted efficiency (*Eff*) and the binary efficiency (*Eff_bin_*)**

We measured the functioning of the network during the nodes removal process using the network efficiency^1-4^. The efficiency measurement is based on the shortest paths notion^5^. We adopt the efficiency (*Eff*) as weighted measure of the network functioning comparing the outcomes with the ones obtained with the binary-topological and widely used largest connected cluster (*LCC*)^1-7^. Here we explain step by step how to compute the efficiency measure.

Let G be a binary network of *N* nodes and *L* links, it can be represented by an *N* × *N* binary adjacency matrix A, where the element *a_ij_*=1 if there is a link between nodes *i* and *j* and 0 otherwise. A weighted network G_w_ can be represents by a *N × N* matrix W where elements *w_ij_*>0 if there is a links of weight *w* between nodes *i* and *j, and wi,j*=0 otherwise (Figure S1).


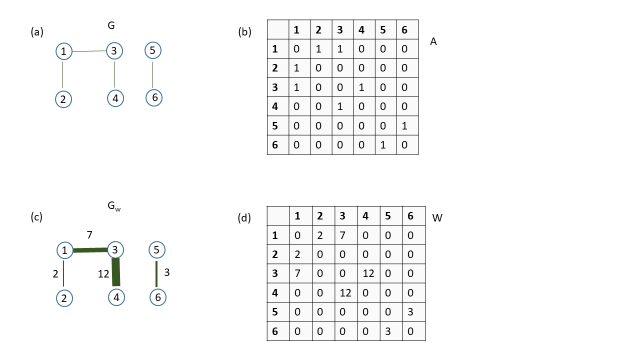


**Figure S1**: (b) *N × N* adjacency matrix A of the binary network G in (a) with no weights associated to the links; (d) *N × N* matrix W of the weighted networks G_w_ in (c) including the weight of the links.

In a binary network G, a path between a couple of nodes is an integer number indicating the amount of links necessary to travel from a node to the other. Thus, in a binary network G the shortest path between nodes *i* and *j* is the minimum number of links necessary to travel between the nodes.

To compute the shortest path in a weighted network G_w,_ we first compute the inverse of the weights of the links. This is a standard procedure with the aim to reduce the length of the links with higher weight and increase the length of the links with lower weight, meaning that nodes joined by links with higher weight are more close to each other. For this reason, a weighted path between a couple of nodes is the sum of the inverse of the weight of links necessary to travel between the couple of nodes; as a consequence, the weighted shortest path is the minimum of the weighted path between the nodes.

Let *p_ij_* the ensemble of all paths between nodes *i* and *j* (either binary or weighted), the shortest path *s_ij_* between the couple of nodes is:

(2)

where 0 < *s_ij_ <*$\infty$if there is a path between nodes *i* and *j*. If there is no path between the couple of nodes, e.g. nodes belong to different isolated clusters, we define *s_ij_*=$\infty$. The elements *s_ij_* belong to the *N × N* shortest paths matrix S of the network. In Figure S2 we depict the binary network with shortest paths matrix S. In Figure S3 we depict the weighted network and the relative shortest paths matrix S.


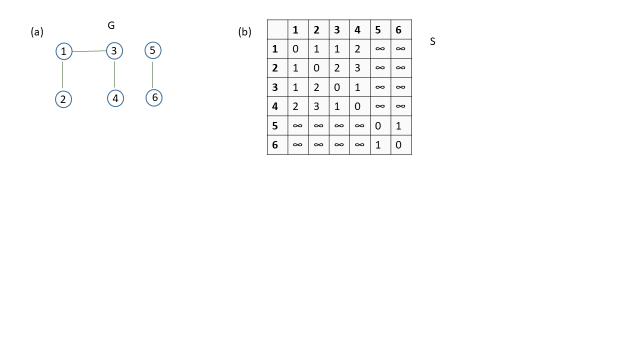


**Figure S2**: (b) *N × N* shortest paths matrix S of the binary network G in (a). Each elements *s_i,j_* in the matrix S represents the shortest path between nodes *i* and *j*. For example, travelling from node 2 and node 4 it is necessary pass throughout at least 3 links, e.g. the element *s_2,4_*=3. Where there is infinitive value, means there is not path between the couple of nodes and the nodes belong to isolate clusters.

**
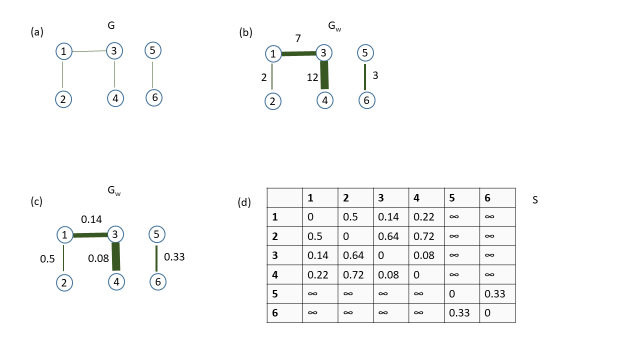
**

**Figure S3**: (a) binary network G; (b) weighted network G_w_; (d) *N × N* shortest paths matrix S of the inverse weight of the weighted network G_w_ in (c). Each elements *s_i,j_* in the matrix S represents the shortest path between nodes *i* and *j*. For example, travelling from node 2 and node 4 it is necessary pass throughout the link weights {0.5,0.14,0.08}, e.g. the element *s_2,4_*=0.5+0.14+0.08=0.72. The infinitive value means there is not path between the couple of nodes and the nodes belong to different clusters.

After having computed the shortest path between a couple of nodes, we can define the efficiency measure between the two nodes as the inverse of the shortest path. Let be *s_ij_* the shortest path between the nodes *i* and *j*, the efficiency *e_i,j_* the nodes is:

 (3)

where *e_ij_*=0 in the case there is no path between nodes *i* and *j* ($\frac{1}{\infty}$, minimum efficiency), and *e_ij_*<0 otherwise. The elements *e_ij_* belong to the *N × N* efficiency matrix E of the network (See Figure S4).

*
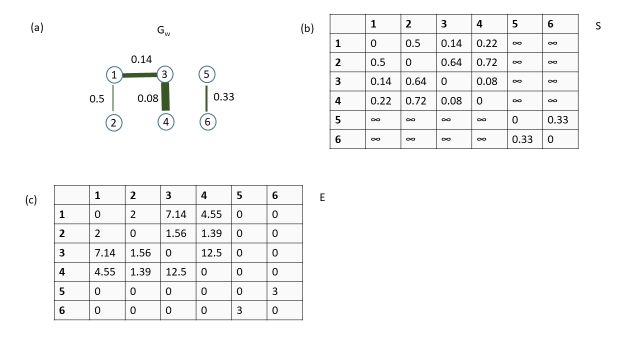
*

**Figure S4**: (a) weighted network G_w_ obtained computing the inverse of the link weight; (b) *N × N* shortest paths matrix S of the weighted network G_w_ in (a). Each elements *s_i,j_* in the matrix S represents the shortest path between nodes *i* and *j*. For example, travelling from node 2 and node 4 it is necessary pass throughout the link weights {0.5,0.14,0.08}, e.g. the element *s_2,4_*=0.5+0.14+0.08=0.72. The infinite value means there is not path between the couple of nodes and the nodes belong to isolate clusters. (c) *N × N* efficiency matrix E of the weighted network G_w_ computed as the inverse of the elements *s_i.j_* in the shortest paths matrix S.

Averaging the efficiency among nodes, we can define the network efficiency *Eff*:

$Eff=\frac{1}{N\left(N-1\right)}\text{ }\sum_{i=1}^{N} \sum_{j=i+1}^{N} e_{i,j}$ (4)

The efficiency computed in Eq. (4) is the (*Eff_bin_*) when computed starting over a binary network G or the weighted efficiency (*Eff*) when computed over the weighted network G_w_ that account the weight of the links.

Computing the reciprocal of the shortest paths means that shorter routes among nodes increase the efficiency of the network and *viceversa*. Further, in the case two nodes are not joined by paths (as in the case of isolated clusters), the shortest path *s_ij_* return the infinite value for the nodes pairs. Thus, computing the reciprocal of the shortest path *s_ij_* for a disjoined couple of nodes *i* and *j* equals zero (lowest efficiency), maintaining the general idea about network efficiency.

Resuming the procedure step by step, to compute *Eff* and *Eff_bin_*, we:

*i*) Compute the reciprocal of each link weight. This way, links with higher weight value represent “wider and faster routes” or, in terms of distance, “shorter routes”, producing lower shortest paths. See Fig. S3 (c) for a representation of the weighted network in Fig. S3 (b).

*ii*) Compute the shortest paths for each node pairs (Eq. 2). See Fig. S4.

*iii*) Compute the efficiency for each pair of nodes by producing the reciprocal of the shortest paths (Eq. 3) (See Fig. S.2 (c)). The higher the shortest paths between two nodes, the lower the efficiency.

*iv*) We sum the efficiency values and we average on the total number of node pairs (i.e. *N*(*N*-1)). The average value of the nodes efficiency is the network weighted efficiency (*Eff* if the network is weighted or *Eff_bin_* if it is binary) (Eq. 4).

**The largest connected cluster (*LCC*)**

The *LCC* is also known as giant component of the system and it is the highest number of connected nodes in the network. The largest connected cluster (*LCC*) is the most widely adopted measure for evaluating the vulnerability of the network under attack or failures of nodes and links^1-3^. In literature we can find many different applications of the *LCC*, ranging from the analyses of network robustness to nodes attack^1-5^ to the evaluation of the efficacy of the vaccination strategies in social complex network^7^.

In complex network science a cluster is a subset of connected nodes. Complex networks may present different clusters, e.g. different subsets of nodes connected among them but disjointed with nodes belonging to other clusters. Let be *S_j_* the size of the *j*-th cluster in the network, the *LCC* is the maximum cluster size:

$LCC=\text{ }\max(S_{j})$ (5)

The removal of nodes-links may disconnect nodes belonging to the giant component thus producing the *LCC* measure decrease. In Figure S5 we show a simple example of the *LCC* computation during the nodes removal process. The *LCC* decrease indicates a reduced possibility to reach nodes in the network. The *LCC* is a simple indicator evaluating the topological connectedness of the network and it not account the weight of the links joining the nodes. The *LCC* is an indicator of the possibility to reach nodes in the network with no consideration about the magnitude of the links-pathways we have to travel. For this reasons we used it like a binary measure of the network functioning not reflecting the heterogeneity of the link weights.

**Figure S5**: The highest number of connected nodes in the initial network (a) is 8, thus the *LCC*=8. In chart (b) we see the network after the removal of node 2, e.g. the network is fragmented in 3 different clusters and the maximum cluster size is 4 (cluster formed by nodes 5,6,7,8) and the *LCC*=4.

**S.2 Real-world complex networks with modified link weights**

To understand how the heterogeneity in the weight of the links affect the robustness of complex weighted networks we artificially tune the weight of the links maintaining the binary topological structure of these systems. We first randomly assign to each link a weight sorted from the rectangular in the interval (1,*Wmax*) distribution tuning the sole parameter *Wmax* indicating the allowed maximum links weight. Then we randomly assign the weight to the links sorting from the bimodal distribution where links weight can have only two distinct values with equals probability (1,*Wmax*). For both the distributions, we tune *Wmax* using the values {1,2,3,5,7,10,20,50,100,1000,100000}. In the case *Wmax*=1 all the links have weight=1 (higher network homogeneity) and the network turn to be a simple binary structure; for *Wmax*≥2 the network start to exhibit variance in link weights with the max variance for *Wmax*=100000 (higher network heterogeneity). In this way the binary topological structure of the real-world network is maintained but any pattern of correlation between the topological and the weighted structure is eliminated, making possible to understand how the simple heterogeneity in links weights affects the system robustness.

**S.3. The real-world complex networks robustness: additional results**

**Robustness of the functioning efficiency (*Eff*) with increasing link weights heterogeneity (*Wmax*)**

Increasing *Wmax* we assist to a slight decrease in robustness (*Eff*) for all the nodes removal strategies (Fig. S12) and a sharp decrease for the strongest links removal strategy (*Strong*); on the contrary, the robustness (*Eff*) increases as a function of *Wmax* when weakest links are removed first (Fig. S12). All these trends are more pronounced for the 2 values distribution of link weights (Fig. S12). Since the rectangular distribution in the interval domain (a,b) owns variance V_unif_ =$\frac{1}{12}{(b-a)}^{2}$ and standard error SE_unif_ =$\frac{1}{\sqrt{12}}(b-a)$, whereas the 2values distribution on the same interval presents variance V_2val_=$\frac{1}{4} {(b-a)}^{2}$ and SE_2val_=$\frac{1}{2} (b-a)$, and SE_2val_> SE_unif_  for b≠a, we argue that the faster decrease in robustness (*Eff*) of the 2 values distribution would be caused by the higher heterogeneity (asymmetry) in link weights.

The difference in networks robustness of the efficiency *(Eff*) related to the higher asymmetry of link weights distribution reaches the 15-20% for *First* and *Str* nodes removal, 30% for *Weak* and the 50-70% for *Strong* links removal (Fig. S11). These findings unveil a general pattern where the link weights heterogeneity affect the robustness of the efficiency functioning (*Eff*) of the real-world complex weighted networks by: i) decreasing the robustness (*Eff*) under nodes removal, both random than attack, ii) decreasing the robustness (*Eff*) under random links removal and strong links removal, iii) increasing the robustness (*Eff*) under weak links removal.

**Robustness of the total flow (*TF*) with increasing link weights heterogeneity (*Wmax*)**

The robustness of the total flow (*TF*) remains constant when by increasing *Wmax* when nodes and links are randomly removed (Fig. S13). This indicates that the proportion of the total flow subtracted to the networks does not change increasing the heterogeneity of the link weights. In other word the robustness evaluated by the total flow in the network (*TF*) to the failure of nodes-links (the so called error in the system) of complex weighted networks is not affected by increasing link weights heterogeneity. Very interesting, we observe the same pattern when we attack the most connected nodes and *First* attack strategy intercept roughly the same amount of flowing with increasing *Wmax*. Differently, we find a robustness (*TF*) decrease when attacking nodes with higher strength (*Str* strategy). The *Str* nodes attack strategy selects nodes joined by links with higher weight, and for this reason when increasing the heterogeneity of the link weights this strategy is able to intercept a major amount of flowing in the system, triggering higher *TF* decrease and higher damage.

We find sharp robustness decrease (*TF*) for the strongest links removal strategy (*Strong*); i.e. when the heterogeneity of the link weights increase, removing strongest links deprives the network of a major amount of total flow (Fig. S13). We find sharp robustness increase (*TF*) for the weakest links removal strategy (*Weak*); i.e. when the heterogeneity of the link weights increase, removing weakest links deprives the network of a minor amount of total flow (Fig. S13). In other terms, when measuring the functioning of the network by the total flow, *Strong* links removal strategy becomes more harmful by increasing the heterogeneity in link weights, where *Weak* links removal strategy becomes less harmful by increasing the heterogeneity in link weights.

**Robustness of the real-world complex networks and the degree-strength coupling**

It has been show that real-world networks showed a non-trivial topology, with degree-strength correlation of the nodes^8-9^. We find that real-world networks exhibit higher efficiency (*Eff*) robustness to the removal of nodes after the link weights randomization (except the Human brain), both random than attack (Fig. S11). See for example Fig. S11 depicting the decrease of the network functioning measures (*Eff* and *TF*) for the first 5 nodes attack; for all the real-world complex weighted networks the randomized counterpart, where weights are randomly re-assigned over the topological structure, showed slower decrease in both the efficiency (*Eff* ) and total flow (*TF*) under nodes attack. This can be explained by the fact that in networks with strength-degree correlation, the removal of higher connected nodes (the so-called hubs) will delete strongest links with higher weight and total flow loss for the system; differently, in the control network, the weights randomization eliminates the correlation and the removal of hubs would intercept less flowing in the network causing a slower decrease in both the efficiency (*Eff*) and the total flow (*TF*). This finding indicates that higher level of nodes degree-strength coupling in real-world complex networks can decrease the robustness of these systems to nodes removal.

**Figure S6**: **Nodes removal outcomes: comparison between *Eff* and *LCC* measurements of complex networks functioning.** Normalized network efficiency (*Eff*) and largest connected cluster (*LCC*) of the real-world networks vs fraction of nodes removal *q* for the nodes attack strategies. The black line: *LCC*, red line: *Eff*.

**Figure S7**: **Nodes removal outcomes: comparison between real-world weights complex networks and randomized weights network controls**. Normalized network efficiency (*Eff*) of the real-world networks vs fraction of nodes removal *q* for the nodes attack strategies. The strategies are black line: real link weights, red line: randomized link weights.

**Figure S8**: **Links removal outcomes: comparison between *Eff* and *LCC* measurements of complex networks functioning.** Normalized network efficiency *Eff* (red line) and largest connected cluster *LCC* (black line) of the real-world networks vs fraction of links removal *q* for the links attack strategies.

**Figure S9**: **Links removal outcomes: comparison between real-world weights complex networks and randomized weights network controls**. Normalized network efficiency (*Eff*) of the real-world complex networks vs fraction of links removal *q* for the links attack strategies. The strategies are black line: real link weights, red line: randomized link weights.

**Figure S10**: **Real-world complex weighted networks functioning decrease under few removals**. The system functioning is normalized by the initial functioning value (e.g. before any removal). For all systems except Human brain network, after an handful of higher strength-degree nodes removal we observe a quick efficiency (*Eff*) and total flow (*TF*) decrease, whereas the binary efficiency (*Eff_bin_*) experienced a slight reduction and the largest connected cluster (*LCC*) remains roughly constant.

**Figure S11**: **Real-world *vs* randomized complex weighted networks functioning under few removals**. The measure of system functioning is normalized by the initial functioning value (e.g. before any removal). We compare the decrease in the functioning measures efficiency (*Eff*) and total flow (*TF*) under 5 node removals between the real-world complex weighted networks and the randomization counterparts. Nodes are removed according to the binary degree (*First*) and the strength (*Str*). The randomized counterpart showed slower functioning decrease than the real-world complex networks, e.g. the randomized networks are generally more robust to nodes attack.

**Figure S12**: **Robustness of real-world networks under increasing heterogeneity of link weights**. The robustness is measured with the efficiency *Eff* of the system functioning under nodes and links removal strategies. Link weights are sorted from rectangular distribution with interval (1,*Wmax*) and from 2 values distribution (1,*Wmax*); the upper limit *Wmax* ranges in (1,10^5^). The outcomes for each *Wmax* value are the average of 10^4^ simulations. **a**, nodes removal strategies**. b**, links removal strategies.

**Figure S13**: **Robustness of real-world networks under increasing heterogeneity of link weights**. The system functioning under nodes and links removal strategies is computed by the total flow *TF* measure. Link weights are sorted from rectangular distribution with interval (1,*Wmax*) and from 2 values distribution (1,*Wmax*); the upper limit *Wmax* ranges in (1,10^5^). The outcomes for each *Wmax* value are the average of 10^4^ simulations. **a**, nodes removal strategies**. b**, links removal strategies.

**Figure S14**: **Robustness of real-world networks under increasing heterogeneity of link weights**. The system functioning under nodes and links removal strategies is computed by the *LCC* measure. Link weights are sorted from rectangular distribution with interval (1,*Wmax*) and from 2 values distribution (1,*Wmax*); the upper limit *Wmax* ranges in (1,10^5^). The outcomes for each *Wmax* value are the average of 10^4^ simulations. **a**, nodes removal strategies**. b**, links removal strategies.

**References**

1. Bellingeri, M. & Cassi, D. Robustness of weighted networks. *Phys. A Stat. Mech. its Appl.* **489,** 47–55 (2018).

2. Latora, V. & Marchiori, M. Efficient Behavior of Small-World Networks. *Phys. Rev. Lett.* **87,** 198701 (2001).

3. Latora, V. & Marchiori, M. Economic small-world behavior in weighted networks. *Eur. Phys. J. B* **32,** 249–263 (2003).

4. Bellingeri, M., Bevacqua, D., Scotognella, F., LU, Z. M. & Cassi, D. Efficacy of local attack strategies on the Beijing road complex weighted network. *Phys. A Stat. Mech. its Appl.* **510,** 316–328 (2018).

5. Newman, M. E. J. The structure and function of complex networks. *SIAM Rev.* **45,** 167–256 (2003).

6. Albert, R. & Barabási, A. Statistical mechanics of complex networks. *Rev. Mod. Phys.* **74,** (2002).

7. Bellingeri, M., Agliari, E. & Cassi, D. Optimization strategies with resource scarcity: from immunization of networks to the traveling salesman problem. *Mod. Phys. Lett. B* (2015).

8. Boccaletti S., Vito, L., Y., M., M, C. & D., H. Complex networks: Structure and dynamics. *Phys. Rep.* **424,** 175–308 (2006).

9. Barrat, A., Barthélemy, M., Pastor-Satorras, R. & Vespignani, A. The architecture of complex weighted networks. *Proc. Natl. Acad. Sci. U. S. A.* **101,** 3747–3752 (2004).

^1,2,11–20,3–10^
